# Exploring Molecular Mechanisms Involved in the Development of the Depression-Like Phenotype in Interleukin-18-Deficient Mice

**DOI:** 10.1155/2021/9975865

**Published:** 2021-10-18

**Authors:** Kyosuke Yamanishi, Masahiro Miyauchi, Keiichiro Mukai, Takuya Hashimoto, Noriko Uwa, Hitomi Seino, Wen Li, Naomi Gamachi, Masaki Hata, Sachi Kuwahara-Otani, Seishi Maeda, Yuko Watanabe, Hiromichi Yamanishi, Hideshi Yagi, Haruki Okamura, Hisato Matsunaga

**Affiliations:** ^1^Department of Psychoimmunology, Hyogo College of Medicine, Nishinomiya, Hyogo 663-8501, Japan; ^2^Department of Neuropsychiatry, Hyogo College of Medicine, Nishinomiya, Hyogo 663-8501, Japan; ^3^Department of Anatomy and Cell Biology, Hyogo College of Medicine, Nishinomiya, Hyogo 663-8501, Japan; ^4^General Education Center, Hyogo University of Health Sciences, Chuo-ku, Kobe, Hyogo 650-8530, Japan; ^5^Hirakata General Hospital for Developmental Disorders, 2-1-1, Tsudahigashi, Hirakata, Osaka 573-0122, Japan

## Abstract

Interleukin-18 (IL-18) is an inflammatory cytokine that has been linked to energy homeostasis and psychiatric symptoms such as depression and cognitive impairment. We previously revealed that deficiency in IL-18 led to hippocampal abnormalities and resulted in depression-like symptoms. However, the impact of IL-18 deficiency on other brain regions remains to be clarified. In this study, we first sought to confirm that IL-18 expression in neural cells can be found in human brain tissue. Subsequently, we examined the expression of genes in the prefrontal cortex of *Il18*^−/−^ mice and compared it with gene expression in mice subjected to a chronic mild stress model of depression. Extracted genes were further analyzed using Ingenuity® Pathway Analysis, in which 18 genes common to both the chronic mild stressed model and *Il18*^−/−^ mice were identified. Of those, 16 were significantly differentially expressed between *Il18^+/+^* and *Il18*^−/−^ mice. We additionally measured protein expression of *α*-2-HS-glycoprotein (AHSG) and transthyretin (TTR) in serum and the brain. In the prefrontal cortex of *Il18*^−/−^ mice, TTR but not AHSG was significantly decreased. Conversely, in the serum of *Il18*^−/−^ mice, AHSG was significantly increased but not TTR. Therefore, our results suggest that in IL-18-deficit conditions, TTR in the brain is one of the mediators causally related to depression, and AHSG in peripheral organs is one of the regulators inducing energy imbalance. Moreover, this study suggests a possible “signpost” to clarify the molecular mechanisms commonly underlying the immune system, energy metabolism, neural function, and depressive disorders.

## 1. Introduction

Major depressive disorder (MDD) is common and increasing in prevalence worldwide, which makes it an urgent health issue. MDD is known to interfere with people's day-to-day activities such as going to work, and these patients report more negative social interactions [1]. In addition, depressive symptoms might make patients more sensitive in everyday experiences of both social acceptance and rejection [2]. Therefore, MDD affects patients' daily life and social interactions.

Immunological dysfunction has been proposed to be an etiological mechanism for MDD, based on meta-analyses; serum interleukin- (IL-) 6, tumor necrosis factor (TNF)-*α*, and IL-18 are increased in MDD patients [3, 4]. Furthermore, with respect to the brain areas responsible, growing evidence supports an association of the prefrontal cortex (PFC), hypothalamus, and hippocampus with MDD [5–8]. In clinical studies, reduced volumes of the PFC and hippocampus were revealed by neuroimaging [7, 9], and abnormal activity of the hypothalamic-pituitary-adrenal (HPA) axis in depressed patients has also been reported [10]. Moreover, the HPA axis and hippocampal homeostasis were shown to be important to prevent the progression of depression [11]. Even though these findings seem to support the possible biological mechanisms of MDD, including the principal brain regions involved [12], the etiological mechanism underlying the development of MDD remains unclear.

IL-18 was initially purified as a proinflammatory cytokine that induces interferon- (IFN-) *γ* [13]. IL-18 is secreted in an active 18 kDa mature form, metabolized by cleaved caspase-1 from a nonactive 24 kDa precursor [14–17]. IL-18 is associated with energy metabolism and psychiatric disorders such as depression [18–20]. Evidence for a role in energy metabolism has been demonstrated in IL-18-deficient mice, which presented diabetes mellitus and dyslipidemia, which leads to nonalcoholic fatty liver disease and steatohepatitis [18, 19, 21]. In the central nervous system, a deficit in IL-18 has been shown to induce hippocampal abnormalities and result in a depressive-like behavioral change [20]. Thus, IL-18 is closely associated with the immune system, energy balance, neural function, and depressive disorders.

In our previous study, these biological roles of IL-18 were intensively examined. In the central nervous system, IL-18-deficient mice showed significantly impaired learning and memory function and exhibited lower motivation, which resulted in depressive-like behaviors. However, only the hippocampal function was evaluated [20].

Hence, we herein sought to determine the molecular expression based on the development of depression in the condition of IL-18 deficiency, especially focusing on the PFC. To do so, we designed a four-step study: (1) confirming IL-18 protein expression in human neurons, (2) comparing gene expression in IL-18-deficient mice with that of the mouse chronic mild stress (CMS) model of depression, (3) evaluating differences in gene expression between the PFC of *Il18*^−/−^ and *Il18*^+/+^ mice by reverse transcription quantitative polymerase chain reaction (RT-qPCR), and (4) measuring the protein expression encoded by identified genes in serum, the PFC, hypothalamus, and hippocampus of *Il18*^+/+^ and *Il18*^−/−^ mice.

## 2. Materials and Methods

### 2.1. Animals

*Il18* ^−/−^ male mice were generated on a C57Bl/6 background as previously described [22]. As controls, we used C57Bl/6 *Il18*^+/+^ male littermates. Mice were housed in groups of three to five in polycarbonate cages with free access to water and standard food in a colony room maintained under controlled conditions as follows: temperature (22 ± 1°C), humidity (50%–60%), and a 12 h light/dark cycle (lights on at 08:00 am). Mice were sacrificed at 12 weeks of age at 10:00 am. Twelve and six *Il18*^+/+^ and *Il18*^−/−^ mice, respectively, were used for RT-qPCR and western blotting, and an enzyme-linked immunosorbent assay (ELISA) was performed using serum samples (*n* = 18 for each). In total, eighteen *Il18*^+/+^ and *Il18*^−/−^ mice were used in the present study. The numbers of mice in this study were decided based on our previous study [20, 23], and the PFC extracted in our previous research was used in the current study to minimize the number of mice sacrificed. All animal experiments were conducted according to the Guide for the Care and Use of Laboratory Animals published by the National Institutes of Health, and all protocols were approved by the Animal Care Committee of Hyogo College of Medicine (Hyogo, Japan; approval nos. A09-206, #28041, #16-013, and #19-046).

### 2.2. Sample Collection

Mice were euthanized by deep anesthesia using 5% isoflurane inhalation (overdose) for at least 5 min. After confirming shallow or absent breathing or a lack of response to pain stimulation, blood from their heart was collected immediately. The serum and brain regions (PFC, hypothalamus, and hippocampus) were removed as previously described, immediately placed in liquid nitrogen, and stored at −80°C until required [24, 25].

### 2.3. Human Brain Tissue

Human paraffin tissue sections from the PFC of healthy individuals (cat. no. T2234051) and depressed patients (cat. no. T2236051DEP) were purchased from BioChain Institute Inc. (Newark, CA, USA). All human tissues in BioChain Institute Inc. were collected after receiving approval from the Institutional Review Board of BioChain Institute Inc. (CA, USA). Moreover, the human study was reviewed by the Ethics Committee and Institutional Review Board of Hyogo College of Medicine (Hyogo, Japan), who confirmed that this study was fully ethically compliant following the clinical research guidelines published on February 28, 2017, by the Ministry of Health, Labor and Welfare and the Ministry of Education (Japan).

### 2.4. Immunofluorescence Staining

Immunofluorescence staining was performed to assess the expression of IL-18 and microtubule-associated protein 2 (MAP2), a mature neuron marker, in the human PFC. Human brain sections were deparaffinized and incubated in HistoVT One antigen retrieval buffer at 90°C for 40 min (cat. no. 06380-76; Nacalai Tesque Inc. Kyoto, Japan). The sections were blocked with 5% bovine serum albumin in phosphate-buffered saline (PBS) for 1 h at room temperature. They were then incubated in Can Get Signal™ immunoreaction enhancer solution (cat. no. NKB-601; Toyobo Co. Ltd., Tokyo, Japan) with primary antibodies overnight at 4°C. The primary antibodies included anti-mouse IL-18 (dilution 1 : 50, cat. no. 60070-1-lg; Proteintech Japan, Tokyo, Japan) and anti-rabbit MAP2 (dilution 1 : 500, cat. no. AB5622; Merck Millipore, Burlington, MA, USA). Anti-mouse IL-18 antibody specificity was first validated by western blot using HeLa whole cell lysates (cat. no. sc-2200; Santa Cruz Biotechnology, Inc., Heidelberg, Germany) as a positive control in accordance with the manufacturer's protocol and previous studies [26, 27]. For immunofluorescence, fluorochrome-conjugated goat anti-mouse IgG H&L secondary antibody (dilution 1 : 200, Alexa Fluor® 488, cat. no. A-11011, Thermo Fisher Scientific K.K., Tokyo, Japan) or goat anti-rabbit IgG H&L secondary antibody (dilution 1 : 1000, Alexa Fluor® 555, cat. no. ab150078; Abcam, Cambridge, UK) was added and incubated for 2 h at room temperature. After washing with PBS, sections were cover-slipped with Vectashield mounting medium with 4,6-diamidino-2-phenylindole (DAPI) (cat. no. H-1800; Vector Laboratories, Inc., Burlingame, CA, USA). The sections were visualized with a fluorescence microscope using red and green filters and blue light (LSM780, Carl Zeiss Co. Ltd., Tokyo, Japan). The images were scanned using ZEN Imaging Software (Carl Zeiss Co. Ltd.) for analysis.

### 2.5. Molecular Analysis

Microarray data from the PFC of CMS model mice [28] and from the hippocampus of *Il18*^−/−^ mice were obtained in our previous studies [20, 24]. Their series entries are GSE49867 and GSE108485, respectively. Molecules showing expression increases of more than twofold or decreases of more than half were identified and then further narrowed down to those expressed in common between the mouse models.

### 2.6. Ingenuity Pathway Analysis (IPA)

IPA (QIAGEN Digital Insights, Redwood City, CA, USA) was used to search for functions of extracted genes as previously described [23]. The molecules identified in the microarray results were further limited to those related to psychiatric diseases (depression, dementia, Alzheimer's disease (AD), and cognitive impairment) as suggested by observing the behavior of *Il18*^−/−^ mice. Core analysis was performed to analyze the functions of these molecules as follows: tissue, brain; all other settings, default. The network explorer of IPA was run with default settings to detect molecule-molecule interactions and to reveal pathways between molecules.

### 2.7. RNA Purification

Total RNA was extracted and purified as previously described, with minor modifications [19]. Briefly, RNA was extracted using ISOGEN (cat. no. 311-02501, Wako Pure Chemical Industries, Ltd., Osaka, Japan), following the manufacturer's protocol, and then incubated with 5 U of RNase inhibitor and 5 U of DNase I at 37°C for 30 min. After phenol/chloroform purification and ethanol precipitation, RNA was dissolved in sterilized and deionized distilled water.

### 2.8. RT-qPCR

RT-qPCR was performed as previously described [19, 23]. Briefly, total RNA (10 ng/reaction) was used in the RNA-direct SYBR Green Real-Time PCR Master Mix: One-step qPCR kit (Toyobo Co. Ltd.) according to the manufacturer's protocol. Samples were examined in duplicate reactions in 384-well plates. The median threshold cycle values were used to calculate fold changes between the groups. The following cycling conditions were used: 30 s at 90°C and 20 min at 61°C for reverse transcription, followed by 45 cycles of 98°C for 1 s, 67°C for 15 s, and 74°C for 35 s. Fold-change values were normalized to *β*-actin (*Actb*) levels using the relative standard curve method. Primer sequences for RT-qPCR are shown in Table 1.

### 2.9. Western Blotting

Protein was extracted from mouse PFC, hypothalamus, and hippocampus as previously described [19]. Briefly, samples were denatured in Laemmli sample buffer for 5 min at 95°C, electrophoresed in a 10%–20% sodium dodecyl sulfate polyacrylamide gel, and transferred onto polyvinylidene difluoride membranes (Hybond-P, Amersham Bioscience, Little Chalfont, UK). The primary antibodies were as follows: monoclonal rabbit anti-*β*-actin (ACTB; cat. no. 5125S; Cell Signaling Technology, Inc., Danvers, MA, USA) as a loading control, polyclonal rabbit anti-*α*2-HS-glycoprotein (AHSG; dilution 1 : 250; cat. no. #bs-2922R; BIOSS, Beijing, China), and polyclonal rabbit anti-transthyretin (TTR; dilution 1 : 200; cat. no. sc-13098; Santa Cruz Biotechnology, Inc., Dallas, TX, USA). Membranes were blocked with 5% skim milk in PBS containing 0.1% Triton X-100, incubated with primary antibodies at 4°C overnight, and then incubated with horseradish peroxidase-conjugated secondary antibodies (#NA9340V and #RPN1025; GE Healthcare, Amersham, UK). For only hypothalamus protein, the same membrane was reused for protein detection. AHSG or TTR was detected first. Then, stripping buffer (Restore Western Blot Stripping Buffer, cat no. 21059, Thermo Fisher Scientific K.K.) was applied, and ACTB was detected.

### 2.10. ELISA

AHSG, IL-18, and TTR in mouse serum were measured by ELISA. ELISA kits for AHSG, IL-18, and TTR were purchased from R&D Systems, Inc. (cat. no. MFTA00; Minneapolis, MN, USA), Medical & Biological Laboratories Co., Ltd. (cat no. 7625; Nagoya, Aichi, Japan), and Immunology Consultants Laboratory, Inc. (cat. no. E-90PRE; Portland, OR, USA), respectively. ELISAs were performed following the manufacturers' protocols.

### 2.11. Statistical Analysis

All results are expressed as means ± standard deviation (SD) and analyzed using Sigmaplot™ software (version 11.0; Systat Software Inc., San Jose, CA, USA). ELISA, RT-qPCR, and western blot data were analyzed by Student's *t*-test or the Mann–Whitney *U* test after a normality test. Differences were considered statistically significant when *P* < 0.05.

## 3. Results

### 3.1. IL-18 Location in the PFC

**Figure 1:**
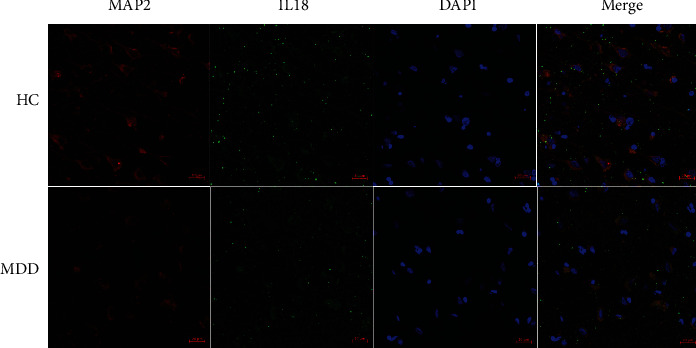
Interleukin- (IL-) 18 expression in the prefrontal cortex (PFC) of a healthy control and a major depressive disorder patient. Immunofluorescence staining was used to evaluate IL-18 expression in the PFC. IL-18 (green), microtubule-associated protein 2 (MAP2) (red), 4,6-diamidino-2-phenylindole (DAPI) (blue), and merged pictures are shown. Cells positive for both IL-18 and MAP2 were present in the human PFC. Scale bars represent 20 *μ*m. HC: healthy control; MDD: major depressive disorder.

To clarify whether IL-18 is expressed in neurons, we performed double immunofluorescence staining in human PFC brain tissue sections. We found IL-18- and MAP2-positive cells coexisted in human PFC, as shown in Figure 1. Validation of the anti-IL-18 antibody was confirmed using the positive control following the manufacturer's protocol (data not shown).

### 3.2. Identification of Molecules Commonly Expressed in CMS and *Il18*^−/−^ Mice

**Figure 2:**
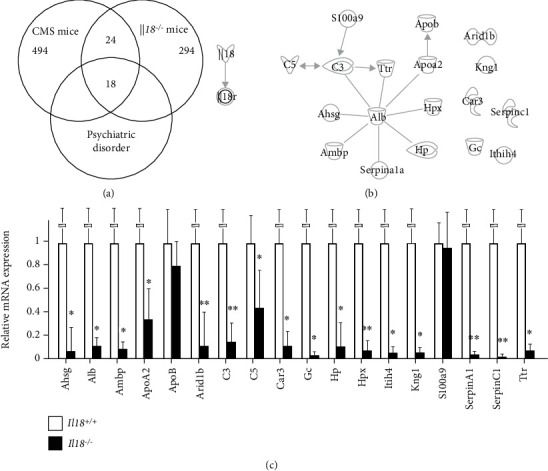
Identification of genes related to psychiatric disorders expressed in both chronic mild stress (CMS) and *Il18^−/−^* mice. (a) Summary of the microarray analyses of CMS mice and *Il18*^−/−^ mice. “CMS mice” indicate the number of genes in the prefrontal cortex (PFC) with expression twofold higher or 0.5-fold lower than in controls. “*Il18*^−/−^ mice” are shown similar to “CMS mice” except the number indicates hippocampal genes. A total of 42 molecules were commonly expressed, of which 18 were related to psychiatric disorders such as depression. (b) Pathway analysis among the 18 extracted genes and interleukin- (IL-) 18 showing that no association was found. (c) mRNA expression of these genes by quantitative reverse transcription polymerase chain reaction (*n* = 12 per group); ^∗^*P* < 0.05 and ^∗∗^*P* < 0.01. The genes are listed in Table 1.

We previously isolated a total of 494 and 294 genes whose expression was increased more than twofold or decreased by more than half from CMS and *Il18*^−/−^ mice, respectively [20, 24]. To examine which genes were responsible for causing depression under IL-18-deficit conditions, we searched for molecules expressed in both CMS and *Il18*^−/−^ mice. We detected 42 such genes, of which 18 encode molecules shown by IPA to be associated with psychiatric disorders. The summarized results are shown in Figure 2(a), and detailed gene information including primer sequences is shown in Table 1.

### 3.3. Disease Association, Functional Annotation, and Interaction among 18 Extracted Genes

We performed IPA core analysis to further analyze the disease associations and functional annotations of the extracted genes (Table 2). Pathway analysis was then performed to elucidate interactions among these genes (Figure 2(b)). Several molecules were shown to be related to each other; however, we observed no interactions between *Il18* and these molecules.

### 3.4. Relative mRNA Expression of 18 Extracted Genes in the PFC of *Il18*^−/−^ and *Il18*^+/+^ Mice

To confirm mRNA expression of the extracted genes in the PFC of *Il18*^−/−^ and *Il18*^+/+^ mice, we performed RT-qPCR. As shown in Figure 2(c), *α*2-HS glycoprotein (*Ahsg*), albumin (*Alb*), alpha-1-microglobulin/bikunin precursor (*Ambp*), apolipoprotein A2 (*ApoA2*), AT-rich interaction domain 1B (*Arid1b*), complement C3 (*C3*), complement C5 (*C5*), carbonic anhydrase 3 (*Ca3*), GC vitamin D binding protein (*Gc*), haptoglobin (*Hp*), hemopexin (*Hpx*), inter-alpha-trypsin inhibitor heavy chain 4 (*Itih4*), kininogen 1 (*Kng1*), serpin family A member 1 (*SerpinA1*), serpin family C member 1 (*SerpinC1*), and transthyretin (*Ttr*) expression was significantly lower in *Il18*^−/−^ mice than in *Il18*^+/+^ mice (*n* = 12 per group, *P* < 0.05).

### 3.5. Protein Expression of AHSG and TTR in the PFC, Hypothalamus, and Hippocampus of *Il18*^−/−^ and *Il18*^+/+^ Mice

**Figure 3:**
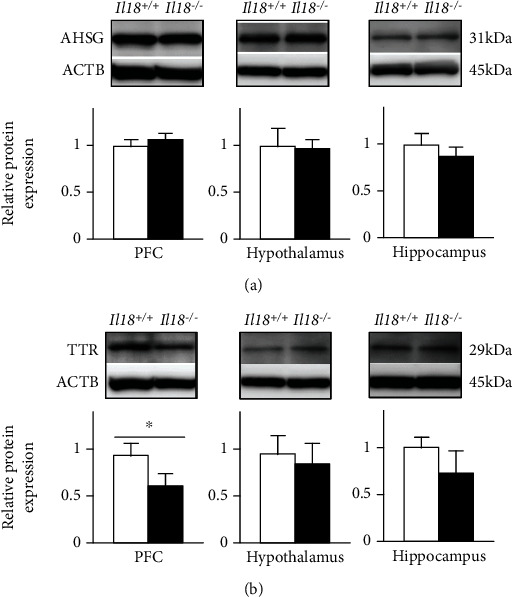
Expression of the *α*-2-HS-glycoprotein (AHSG) and transthyretin (TTR) proteins in the brains of *Il18*^−/−^ and *Il18*^+/+^ mice. Representative immunoblots showing the expression levels of (a) AHSG and (b) TTR in the prefrontal cortex (PFC), hypothalamus, and hippocampus of *Il18*^−/−^ and *Il18*^+/+^ mice. (a) AHSG expression did not differ significantly. (b) TTR expression in the PFC of *Il18*^−/−^ mice was significantly lower than in *Il18*^+/+^ mice. For the hypothalamus, ACTB expression was measured after stripping the blot after detection of TTR or AHSG. *n* = 6 per group; ^∗^*P* < 0.05.

RT-qPCR showed that mRNA expression decreased significantly for 16 of 18 molecules in *Il18*^−/−^ mice. In our previous study [25], we proposed that *Ahsg* and S100 calcium binding protein A9 (*S100a9*) were key mediators not only of depression but also of physiological dysregulation, for example, diabetes mellitus. Additionally, *Ttr* was suggested to be an inducer of MDD. Therefore, we focused on the protein expression of AHSG and TTR, which showed significant differences in mRNA expression between *Il18*^−/−^ and *Il18*^+/+^ mice. Contrary to our expectations, only TTR expression in the PFC of *Il18*^−/−^ mice was significantly decreased compared with *Il18*^+/+^ mice, whereas AHSG expression was not changed (*n* = 6 per group, *P* < 0.05, Figures 3(a) and 3(b)).

### 3.6. Protein Expression of AHSG and TTR in the Serum of *Il18*^−/−^ and *Il18*^+/+^ Mice

**Figure 4:**
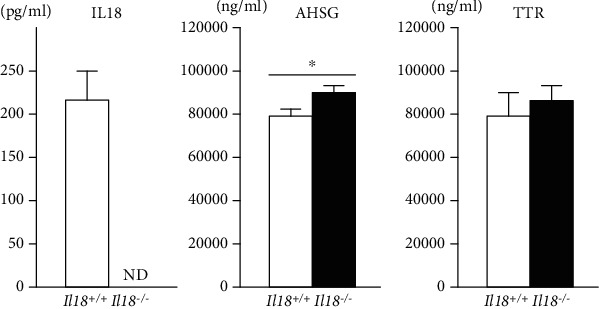
Expression of *α*-2-HS-glycoprotein (AHSG), interleukin- (IL-) 18, and transthyretin (TTR) in the serum of *Il18*^−/−^ and *Il18*^+/+^ mice. Serum IL-18 levels were not detected in *Il18*^−/−^ mice (ND: not detected; *n* = 18 per group). Serum AHSG and TTR protein expression was measured to compare the central nervous system and peripheral response. In *Il18*^−/−^ mice, serum AHSG levels, but not TTR, were significantly higher than in *Il18*^+/+^ mice. *n* = 10 per group; ^∗^*P* < 0.05.

The serum level of IL-18 was not detected in *Il18*^−/−^ mice (*n* = 18 per group, Figure 4). To test our hypothesis that AHSG and TTR could be markers linked to MDD and the dysregulation of physiological homeostasis, we measured serum AHSG and TTR levels in *Il18*^−/−^ and *Il18*^+/+^ mice. Serum AHSG, but not TTR, was significantly higher in *Il18*^−/−^ mice than in *Il18*^+/+^ mice (*n* = 10 per group, *P* < 0.05, Figure 4).

## 4. Discussion

In this study, we profiled the function of IL-18 in the central nervous system as follows: (1) IL-18 was found in the cytoplasm of human neurons; (2) 16 genes expressed in the PFC of *Il18*^−/−^ mice were shared with a CMS model of depression; (3) TTR in the brain, including the PFC, was a mediator associated with depression in IL-18 deficiency; and (4) AHSG in the serum was associated with the dysregulation of physiological homeostasis, such as energy imbalance or immune impairment.

IL-18 is a proinflammatory cytokine promoting IFN-*γ* production that is secreted by Th1 immune cells when exposed to lipopolysaccharide stimulation [13]. Therefore, in the central nervous system, microglia can secrete IL-18 [29]. In the human brain, *IL18* gene expression in the PFC has been examined previously; however, its protein expression has not [30]. Figure 1 shows the costaining of IL-18 and MAP2 in the PFC of both a healthy control and an MDD patient. Thus, our results showed the existence of a precursor form of IL-18 with a molecular weight of approximately 24 kDa in neural cells, suggesting that IL-18 might play a role in neurons of the PFC.

From the results of the core analysis shown in Table 2, we identified *C3*, *C5*, *Kng1*, and *Ttr* as genes showing decreased expression in the PFC of *Il18*^−/−^ mice compared with *Il18*^+/+^ mice (Figure 2(c)). C3 is one of the main factors of inflammatory signaling pathways that promotes IL-1*β* secretion through activation of the NLR family pyrin domain-containing 3 inflammasome, including IL-18 [31]. The complement system exerts some effect on the central nervous system, and its impairment might induce the progression of neurodegenerative disorders such as AD [32]. Moreover, clinical studies have found reduced expression of C3 in both the serum and cerebrospinal fluid (CSF) of MDD patients [33, 34]. In an animal model of AD with C3 deficiency, increased amyloid-*β* (A*β*) plaque and neurodegeneration in the PFC and hippocampus were observed, suggesting that cerebral C3 may play an important role in inhibiting the progression of AD [35]. As shown in Table 1 and Figure 2(c), *C3* expression was decreased in the PFC of *Il18*^−/−^ mice but increased in the hippocampus.

With regard to *C5*, a clinical study revealed that CSF C5 expression was significantly increased in MDD patients, resulting in microglial neuroinflammation [36]. Furthermore, bradykinin, a final product of *Kng1*, may be associated with the emergence of depression [37]. Decreased bradykinin is thought to reduce brain-derived neurotrophic factor release, inhibit synaptogenesis, and enhance the progression of depression [37]. Even though the role of these two molecules in the central nervous system remains unclear, they might connect depression with neuroinflammation. In sum, these immune-related molecular changes suggest that an IL-18 deficiency causes the abnormal activation of immune pathways and affects neural plasticity, leading to psychiatric disorders such as MDD and AD, though other inflammatory factors should be considered.

Disruption of the blood-brain barrier (BBB) might also be a crucial factor in depression with some immune impairment [38, 39]. *C3a* and bradykinin are vasoactive molecules that disrupt the BBB [40], while IL-18 helps to mitigate such disruption [41]. AHSG might inhibit the brain inflammatory response in the early stage of cerebral ischemic injury, resulting in the disruption of the BBB [42]. TTR has an ability to cross the BBB from the brain to the blood; however, it cannot cross in the opposite direction [43]. Therefore, TTR might play a role in removing the peptide from the brain and inhibiting the reverse flow [43]. We detected reduced *C3* and *Kng1* mRNA expression, decreased TTR protein in the PFC, and increased AHSG expression in the serum in *Il18*^−/−^ mice compared with *Il18*^+/+^ mice (Figure 2(c)). These results indicate that IL-18 deficiency might influence the BBB function via *C3* and *Kng1*, leading to the development of a depressive phenotype, although AHSG might protect against neuroinflammation under a depressive state.

In a clinical study, serum IL-18 levels of depressed patients were increased compared with healthy controls [44], while decreased C3 in the CSF of depressed patients suppressed inflammasome activation and decreased IL-18 [31]. Therefore, our results suggest that increased IL-18 may be a compensatory response in MDD patients, and that IL-18 may have protective roles against inflammation in the brain of MDD patients. Furthermore, these findings also indicate that neuroinflammation is a major mechanism involved in the progression of MDD.

AHSG is secreted into the blood mainly by the liver and is also expressed in the cerebral cortex, thalamus, and hippocampus [45, 46]. Based on previous studies, AHSG functions in the induction of inflammation and energy balance [47, 48]. During the inflammatory response, AHSG synthesis is downregulated by IL-6, TNF-*α*, and IFN-*γ* [49, 50]. IL-18 has a role in IFN-*γ* induction; therefore, increased serum AHSG levels might be caused by reduced IFN-*γ* following IL-18 deficiency, as shown in Figure 4.

In energy homeostasis, several studies have reported a relationship between AHSG and the glucose and lipid balance. Indeed, higher plasma AHSG levels were linked to higher insulin resistance and fat accumulation in the liver [51]. Moreover, increased serum AHSG was correlated with the risk of metabolic syndrome, including higher triglycerides [48]. *Il18*^−/−^ mice have been reported to show higher insulin resistance, diabetes mellitus, obesity, and dyslipidemia [18, 19, 21], and their serum AHSG levels were higher than those of *Il18*^+/+^ mice (Figure 4); hence, our results are consistent with previous studies. Furthermore, higher AHSG was reported in male MDD patients compared with controls [52]. While AHSG expression in the PFC, but not the thalamus and hippocampus, was increased in our depressive model mice, its expression in *Il18*^−/−^ mice was unchanged [25] (Figure 3(b)). Thus, these results suggest that AHSG is a key factor connecting depression, energy homeostasis, and the immune system in IL-18 deficiency.

According to the Allen Brain Atlas Human (https://human.brain-map.org/), TTR is expressed in the PFC, hypothalamus, and hippocampus [53, 54]. In depressed patients, TTR was significantly decreased in the CSF compared with healthy controls [55]. Similar results were obtained in AD studies [56]. TTR was suggested to be involved in A*β* protein degradation and clearance; thus, reduced TTR in the brain may increase A*β* levels and cause AD progression [56]. Moreover, TTR-deficient mice exhibited fewer depressive behavioral changes through increased levels of noradrenaline, but not dopamine or serotonin, in the limbic forebrain, and it was suggested that TTR has an important role in resilience to stress exposure [55, 57]. In our previous study, *Il18*^−/−^ mice showed less motivation and had impaired learning and memory, which are clinically included in depression or dementia [20]. Furthermore, TTR expression in the PFC of *Il18*^−/−^ mice was significantly decreased compared with *Il18*^+/+^ mice in this study (Figure 3(b)). However, serum TTR levels were unchanged (Figure 4). No correlation was observed between serum TTR and CSF in a human study, indicating that TTR expression in the brain may differ from that in peripheral organs [58]. Thus, lower TTR of the PFC in *Il18*^−/−^ mice might be a mediating factor of depression.

Regarding the limitations of our study, because of the limited number of human samples, the immunofluorescence results were insufficient. To validate our hypothesis, additional human samples are required. Second, we discussed the relationship among BBB, depression, and our extracted genes; however, we did not analyze the function of the BBB. Additional experiments will be indispensable to clarify the function of the BBB in IL-18 deficiency. Third, we focused on AHSG and TTR in this study; however, the details of their functions are still unclear. Therefore, mechanistic analysis is needed to explore their functions. Moreover, our study was limited by the fact that we only measured AHSG and TTR in the serum and not in CSF of *Il18*^+/+^ and *Il18*^−/−^ mice. To further examine the validity of our hypothesis, measurements in CSF are warranted.

## 5. Conclusion

In conclusion, we investigated the effects of IL-18 deficiency on the central nervous system and its association with psychiatric disorders. First, we demonstrated the presence of IL-18 in the cytoplasm of human PFC neurons. Then, we identified 16 molecules that might cause the depressive characteristics of *Il18*^−/−^ mice; in particular, decreased TTR in the PFC might be a mediator for developing depression. Finally, AHSG in peripheral organs might also be an inducer of immune and energy imbalance in *Il18*^−/−^ mice. Although these identified molecules are related to several psychiatric disorders such as MDD and AD, the mechanism by which abnormal immune function affects the central nervous system and psychiatric diseases remains unclear. IL-18 might be associated with a potential strategy for elucidating this mechanism and novel treatments for MDD and AD. It might also be a potential “signpost” to clarify the molecular links among the immune system, energy metabolism, neural function, and psychiatric diseases, though further study is required for confirmation.

## Figures and Tables

**Table 1:** Genes identified by ingenuity pathway analysis and their primer sequences.

Symbol	GenBank ID	CMS mice	*Il18* ^−/−^ mice	Primer sequences, 5′-3′ (forward/reverse)
*Ahsg*	NM_013465	3.389	3.053	ACTTGCCATGCTTTGGACCC
				CCTCCACCGCGTGCTCA
*Alb*	NM_009654	3.548	3.582	GAAAGCTGCCTGACCCCGAA
				AGCACTTCATTCTCTGACGGACA
*Ambp*	NM_007443	3.047	3.066	CACCATCACTGCCAAGCTC
				ACATTCAGGGCCACATCCTT
*Apoa2*	NM_013474	3.117	1.529	TACTTTCAGAGCATGACTGATTATGGCAA
				TCGTGTGTCTTCTCAAAGTATGCCT
*Apob*	NM_009693	3.787	1.926	TCTGGGGCATCCATGAAATTATCAACA
				GCAGCTCTCCCATCAAGACT
*Arid1b*	NM_001085355	1.332	-1.104	CCGCCATCTCCTGCTAACTCA
				ACCTCTGCCATCGAATTGCT
*C3*	NM_009778	2.942	1.26	ATCAAAATCCCTCCCAAGTCCTCG
				TCCACCTCTTGTTGGCCGAT
*C5*	NM_010406	2.632	1.555	ATACACTCAAGGCAAAGGTGTT
				TGGATCTGTTCTCCTCGCACA
*Car3*	NM_007606	3.293	1.216	GACCCATCATGCCTGTTCCC
				AGCAGCCACACAATGCACTC
*Gc*	NM_008096	3.229	2.918	TCCTGTGAAAGTGATGCTCCC
				CCATGCAGAGTTTCCGCTCCA
*Hp*	NM_017370	2.93	2.288	TTCTCCACCCCAACCACTCC
				CTCTCGGTTACAAGCACCCTC
*Hpx*	NM_017371	2.969	1.928	TGCACTGCTGTCTGACCATCG
				AGCTATGCCATCCATCACGG
*Itih4*	NM_018746	2.545	1.984	AGAACAAGACCAAGGCTCATATCCG
				CCCATTCAGCACCGTGTCC
*Kng1*	NM_023125	2.995	2.26	AATGCTAACGTGTACATGAGACCT
				TTCTTGCCATTTCAGTCATATCTAATGCTT
*S100a9*	NM_009114	1.195	1.265	AAGCTGCATGAGAACAACCC
				ATGGCTGACCTCTTAATTACTTCCC
*SerpinA1*	NM_009246	3.542	2.886	CCATATCCCCAGACTGTCCA
				CAGCCCCATTGTTGAAGATCCG
*SerpinC1*	NM_080844	3.308	2.425	CGACATCTGCATAGCGAAGCC
				AGCCATCCTCCTCGGT
*Ttr*	NM_013697	3.392	-1.019	ATCTTGCCAAAGCAGTAGCAT
				AACACGGTTTATAGAGCAAGAACACT

“CMS mice” and “*Il18*^−/−^ mice” represented fold change by log_2_ ratio of CMS/control and *Il18*^−/−^/*Il18*^+/+^, respectively.

**Table 2:** Disease association or functional annotation based on ingenuity pathway analysis.

Disease or function annotation	*P* value	Molecules
Disruption of blood-brain barrier	≤0.001	*C3*, *Kng1*
Inflammatory response of the cerebral cortex	0.001	*Kng1*
Diameter of basilar artery	0.001	*Kng1*
Inflammatory response	0.001	*C3*, *Kng1*
Cellular homeostasis	0.001	*C5*, *Kng1*
First-onset paranoid schizophrenia	0.002	*TTR*
Liberation of arachidonic acid	0.002	*Kng1*
Infiltration by CD3-positive T lymphocytes	0.002	*C5*
Accumulation of microglia	0.002	*C5*
Liberation of stearic acid	0.002	*Kng1*
Cellular infiltration of dendritic cells	0.002	*C5*
Thrombus	0.002	*Kng1*
Immune response of the brain	0.002	*C3*, *Kng1*
Size of infarct	0.003	*C3*, *Kng1*
Brain damage	0.005	*C5*, *Kng1*
Damage of blood-brain barrier	0.007	*Kng1*
Abnormal morphology of the hippocampal CA3 region	0.007	*C3*
Concentration of arachidonic acid	0.008	*Kng1*
Alzheimer disease	0.010	*C3*, *Ttr*
Flow of blood	0.016	*Kng1*
Edema of the brain	0.019	*Kng1*
Quantity of amyloid fibrils	0.019	*C3*
Apoptosis	0.020	*C5*, *Ttr*
Damage of brain cells	0.022	*C5*
Permeability of blood-brain barrier	0.022	*Kng1*
Cell viability of granule cells	0.025	*C5*
Disorder of the basal ganglia	0.032	*C3*, *Kng1*
Neurodegeneration of the cerebellum	0.036	*C3*
Volume of infarct	0.045	*Kng1*
Loss of neurons in the central nervous system	0.047	*Ttr*

## Data Availability

The datasets shown and/or analyzed in the present study are available from the corresponding author upon reasonable request.
